# Impact of a wastewater treatment plant on microbial community composition and function in a hyporheic zone of a eutrophic river

**DOI:** 10.1038/srep17284

**Published:** 2015-11-26

**Authors:** Siavash Atashgahi, Rozelin Aydin, Mauricio R. Dimitrov, Detmer Sipkema, Kelly Hamonts, Leo Lahti, Farai Maphosa, Thomas Kruse, Edoardo Saccenti, Dirk Springael, Winnie Dejonghe, Hauke Smidt

**Affiliations:** 1 Laboratory of Microbiology, Wageningen University, Dreijenplein 10, 6703 HB Wageningen, The Netherlands; 2 Flemish Institute for Technological Research (VITO), Separation and Conversion Technology, Boeretang 200, 2400 Mol, Belgium; 3 KU Leuven, Division Soil and Water Management, Kasteelpark Arenberg 20, 3001 Heverlee, Belgium; 4 Department of Veterinary Biosciences, University of Helsinki, PO Box 66, FI-00014, Finland; 5 Laboratory of Systems and Synthetic Biology, Wageningen University, Dreijenplein 10, 6703 HB, Wageningen, The Netherlands

## Abstract

The impact of the installation of a technologically advanced wastewater treatment plant (WWTP) on the benthic microbial community of a vinyl chloride (VC) impacted eutrophic river was examined two years before, and three and four years after installation of the WWTP. Reduced dissolved organic carbon and increased dissolved oxygen concentrations in surface water and reduced total organic carbon and total nitrogen content in the sediment were recorded in the post-WWTP samples. Pyrosequencing of bacterial 16S rRNA gene fragments in sediment cores showed reduced relative abundance of heterotrophs and fermenters such as *Chloroflexi* and *Firmicutes* in more oxic and nutrient poor post-WWTP sediments. Similarly, quantitative PCR analysis showed 1–3 orders of magnitude reduction in phylogenetic and functional genes of sulphate reducers, denitrifiers, ammonium oxidizers, methanogens and VC-respiring *Dehalococcoides mccartyi*. In contrast, members of *Proteobacteria* adapted to nutrient-poor conditions were enriched in post-WWTP samples. This transition in the trophic state of the hyporheic sediments reduced but did not abolish the VC respiration potential in the post-WWTP sediments as an important hyporheic sediment function. Our results highlight effective nutrient load reduction and parallel microbial ecological state restoration of a human-stressed urban river as a result of installation of a WWTP.

Rivers in urban areas are often heavily impacted by untreated wastewater discharge containing excessive organic inputs that can induce anoxia, eutrophication of freshwater ecosystems and severe reduction in water quality^1^. Half of the world’s population lived in urban areas in 2008^2^. This number is predicted to increase to 66% by 2050 mainly due to urban growth in less developed countries^3^, expected to lead to a similar increase in the production of nutrient rich urban waste water. Wastewater treatment plants (WWTPs) are one of the most common measures of modern environmental biotechnology to treat municipal wastewater. Wastewater treatment removes a large fraction of nutrients, before the resulting effluent is discharged into receiving water bodies^4^. WWTP-induced reduction in anthropogenic nutrient input into fresh water ecosystems is in line with the European Water Framework Directive (WDF) that aims at protection of aquatic ecosystems and optimisation of water quality to achieve good ecological and chemical status of these ecosystems^5^. Accordingly, 20–80% of WWTPs in European countries have implemented tertiary treatment that uses technological advances for chemical and biological removal of recalcitrant organic pollutants as well as inorganic nutrients such as nitrogen and phosphorus^6^.

Parallel to reducing nutrient load into downstream receiving ecosystems, complementary measures are mandatory to monitor their ecological state. How will reducing the nutrient concentrations of treated effluent affect the microbial structure and function in the receiving environment ? How will it impact the trophic state of the ecosystem and processes dependent on otherwise high nutrient load ? Allochthonous organic carbon for example fuels river biogeochemical activities^7^, which mainly take place in the riverbed sediment, either at or just below the surface, including the zone where shallow groundwater mixes with surface water known as the hyporheic zone^8^. The high organic matter content in hyporheic zones allows multiple critical reactions in biogeochemical cycling and hence natural attenuation of inorganic and organic pollutants present in upwelling contaminated groundwater plumes^9^. For instance, in recent years, the hyporheic zone has received much attention because of its capacity to retain or degrade groundwater contaminants like chlorinated aliphatic hydrocarbons (CAHs)^10^,^11^,^12^,^13^.

In spite of intensive studies on microbial community composition and function in hyporheic zones^14^,^15^,^16^,^17^,^18^,^19^,^20^,^21^,^22^,^23^,^24^,^25^, far less attention has been paid to the impact of WWTPs effluent on the microbial ecology of riverbed sediments^26^,^27^,^28^. Decreased microbial diversity^26^,^27^ and altered biogeochemical cycling of nitrogen^28^ were reported in downstream effluent impacted sediments compared to samples taken upstream of the WWTP. However, the outcome at other locations may differ from these observations, as the impact of a WWTP on the downstream sediment microbiota depends on different factors such as type of the WWTP and hence the composition of the effluent, the population size of the urban area, climate and geography of the region, the size and flow rate of the river, and the buffering and self-purification capacity of the stream sediments to stresses.

In the present study, we investigated microbial community composition and function across sediment depth in a CAH impacted hyporheic zone of a short stretch of the Belgian Zenne River, as a response to an upstream WWTP installation. The Zenne river was notorious for being one of Belgium’s most polluted rivers receiving untreated municipal effluents from the Brussels Capital Region leading to severe eutrophication of the river. In previous studies performed at that river stretch between 2004 and 2007, during which the Zenne surface water was eutrophic, the highly reducing and organic rich sediments were shown to be conducive to natural attenuation of CAHs present in the discharging groundwater mainly by organohalide respiration (OHR)^12^,^13^,^29^. Since March 2007, a technologically advanced WWTP was installed approximately one km upstream of the test site to treat the municipal wastewater produced by 1.4 million inhabitants prior to its discharge into the river (www.aquiris.be). The biological treatment line of the plant consists of a primary settling stage and a subsequent modern tertiary treatment technology for elimination of nitrogen, phosphorus and carbon-containing pollution by an activated sludge process. The implementation of the WWTP resulted in important chemical changes in the surface water composition such as decrease in organic carbon content and increase in dissolved oxygen concentration. For a conclusive assessment of the impact of the installation of a WWTP on the receiving river ecosystem, we studied the sediment microbial community as indicator for system recovery and impact on particular ecosystem function i.e. vinyl chloride (VC) respiration. In contrast to former studies based on differences in upstream and downstream sediment microbial community where no information was available about the pre-WWTP conditions^26^,^27^,^28^, we sampled our intensively studied test site two years before (2005) and three and four years after (2010 and 2011, respectively) construction of the WWTP.

## Results

### Chemical characterization of the study site

**Figure 1:**
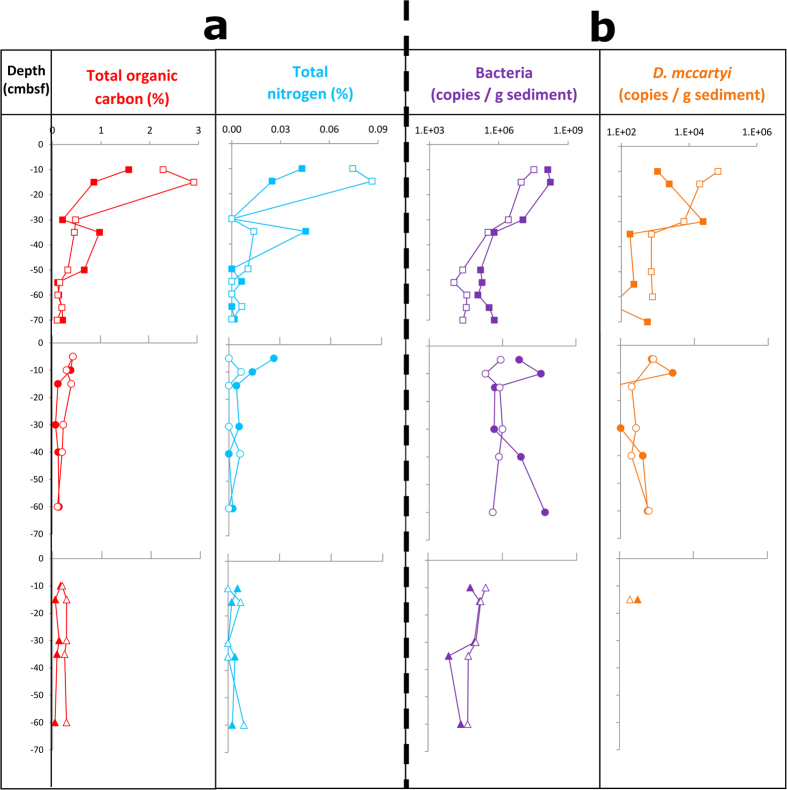
Sediment core characteristics. Total organic carbon and total nitrogen content (**a**) and 16S rRNA gene copy numbers of bacteria and *D. mccartyi* (as measured by qPCR) (**b**) in duplicate (filled (A) and open (B) symbols) sediment core taken in 2005 (top), 2010 (middle) and 2011 (bottom). Each qPCR value represents the average value obtained from triplicate reactions. Sediment-surface water interface was considered as the zero-depth. cmbsf: centimetres below surface.

No obvious trends were observed in surface water temperature, concentrations of anions and cations (except Fe) in the surface water during the experimental period (Table 1): none of the q-values for the Spearman *ρ* correlation was found significant at the 0.25 level after Benjamini-Hochberg correction for multiple testing (Supplementary Table S3). However, dissolved organic carbon (DOC) (*ρ* = −0.89, q-value* *=* *0.09), oxygen (*ρ* = + 0.71, q-value* *=* *0.09), pH (*ρ* = −0.66, q-value* *=* *0.09), Fe (*ρ* = −0.7, q-value* *=* *0.14) and conductivity (*ρ* = −0.52, q-value* *=* *0.24) showed significant differences. In particular we observed that the dissolved oxygen content in the surface water increased to above 3 mg/L in 2007 and on average above 4 mg/L after 2008 (q-value = 0.2, Kruskal-Wallis test). DOC concentrations in the surface water decreased from an average of 51 ± 27 mg/L in the period Dec 2005-May 2006 to an average of 7 ± 1 mg/L in May-Sept 2011 (q-value = 0.24). The DOC and sulphate values in interstitial water (ISW) collected by means of permanent pore water samplers were not significantly different (Table 2). The concentrations of nitrate and nitrite were always below detection limit. The total organic carbon (TOC) significantly decreased with depth (Fig. 1a, Supplementary Table S4,5) in most cores except for core-A in 2010 (*ρ* = −0.83, q-value = 0.29) and core-B in 2011 (*ρ* = 0.29, q-value = 0.87). The total nitrogen (TN) content on the other hand decreased with depth only for core-B in 2005 (*ρ *=* *−0.67, q-value = 0.19) and core-A in 2010 (*ρ *=* *−0.89, q-value = 0.19). A significant decrease over time for both TOC and TN values was observed only in the top 20 cm sediment horizon (Kruskal-Wallis q-values of 0.01 and 0.01, respectively) (Supplementary Table S6).

### Quantification of phylogenetic and functional gene markers in sediment cores

The highest bacterial 16S rRNA gene numbers were detected in surficial layers of both cores taken in 2005 with numbers above 10^7^ copies per gram of sediment (Fig. 1b, Supplementary Table S4). Bacterial 16S rRNA gene numbers in both 2005 cores significantly declined with depth to below 10^5^ per gram of sediment in deeper layers while no significant depth-related reduction was noted in post-WWTP cores (Supplementary Table S5). Bacterial 16S rRNA gene numbers showed a significant reduction over time in post-WWTP cores both in top and bottom sediment horizons (Kruskall-Wallis q-values of 0.01 and 0.07, respectively) (Supplementary Table S6). Similar to the bacterial trends, 16S rRNA gene copy numbers of *Dehalococcoides mccartyi* showed significant reduction with depth in the pre-WWTP core samples (Supplementary Table S5) while reduction over time was only significant in the top sediment horizon (Kruskall-Wallis q-value = 0.01). The abundance of functional gene biomarkers for sulphate reducers (*dsrB*), methanogens (*mcrA*), bacterial/archaeal ammonium oxidizers (bacterial/archaeal *amoA*) and denitrifiers (*nirK*, *nirS*, *nosZ*) significantly decreased over time in post-WWTP (Supplementary Fig. S2, Supplementary Table S6–8).

### Occurrence of VC respiration activity in sediment microcosms

**Figure 2:**
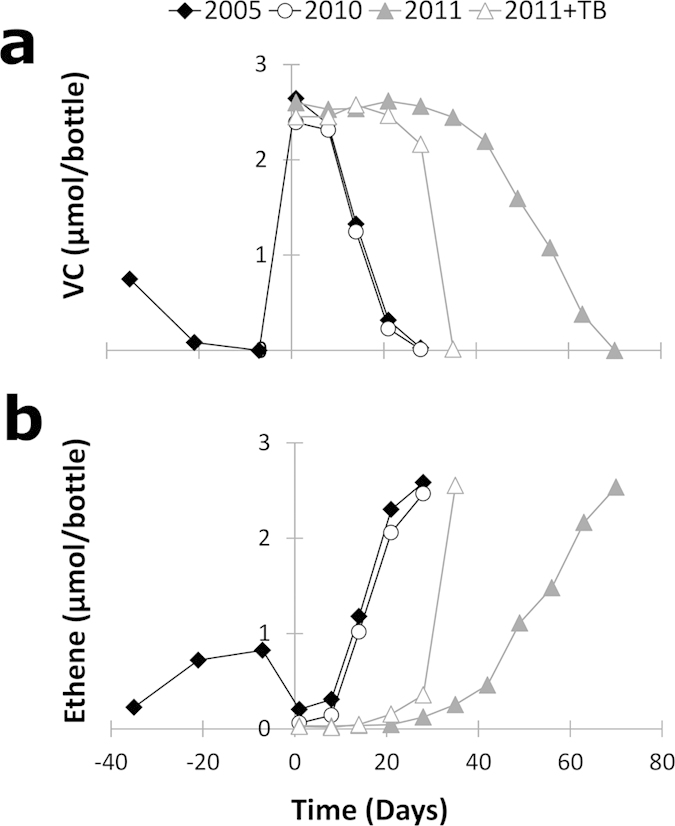
Dechlorination in sediment microcosms. VC dechlorination (**a**) and accumulation of ethene (**b**) in microcosms prepared from top 20 cm riverbed sediment from 2005, 2010, 2011 and 2011 stimulated with tree bark (TB). Sediment bottles of 2005 were re-spiked with VC on day 28 after initial dechlorination of residual VC contamination in the original sample. The day of VC spike was considered as the day zero on the x-axis.

VC respiration was compared between samples taken before and after the installation of the WWTP in microcosms prepared from the top 20 cm sediments and groundwater from the SB1 monitoring well (Fig. 2). VC dechlorination was observed in microcosms containing sediment material sampled in 2005 and in 2010 (Fig. 2a) after seven days and, stoichiometric quantities of ethene were produced within 28 days (Fig. 2b). VC dechlorination in microcosms prepared with sediment material sampled in 2011 started after a lag phase of 35 days, and complete VC dechlorination to ethene was observed after 70 days. Addition of tree bark to sediments from 2011 reduced the lag phase to 14 days, and all VC was stoichiometrically dechlorinated to ethene within 35 days.

### Composition and distribution of the most abundant bacterial taxa

Prior to pyrosequencing, DNA extracts of a total of 80 sediment core slices at 5 cm intervals were analyzed by 16S rRNA gene-based PCR-DGGE bacterial community fingerprinting. Based on the obtained patterns (Supplementary Fig. S1), a total of 40 representative samples were selected for pyrosequencing of the PCR-amplified V1-V2 region of the bacterial 16S rRNA gene. A total of 309191 reads was obtained for all 40 samples. After quality filtering, denoising, and chimera removal, a total of 269411 reads was recovered ranging from 967 to 20335 reads per sample (Supplementary Table S9), with average and median of 6739 and 6068 reads per sample, respectively.

**Figure 3:**
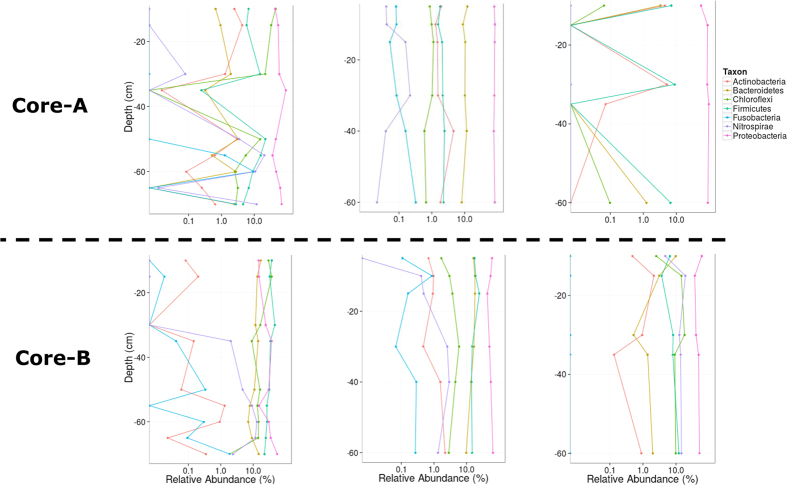
Dominant bacterial phyla. Log-scale relative abundance of the dominant bacterial phyla at different depths in the duplicate sediment core samples taken in 2005 (left), 2010 (middle) 2011 (right). Sediment-surface water interface was considered as the zero-depth.

*Proteobacteria*, *Firmicutes*, *Chloroflexi*, *Bacteroidetes*, *Nitrospirae*, *Fusobacteria,* and *Actinobacteria* were the seven most abundant phyla in all samples (Fig. 3). The *Chloroflexi* phylum was the most abundant in the surface layers of the pre-WWTP samples and generally declined with depth (Fig. 3, right), which was statistically significant in core-B (*ρ *=* *−0.77, q-value = 0.18). In contrast, *Nitrospira* had higher relative abundance in deeper sediment layers (*ρ *=* *0.8, q-value = 0.16). No significant depth-related stratification of bacterial community composition was noted in post-WWTP samples at the phylum level (data not shown). However, significant differences were observed between pre- and post-WWTP samples. The relative abundance of *Chloroflexi* and *Firmicutes* significantly decreased in post-WWTP samples while those of *Nitrospirae* and *Proteobacteria* increased significantly (Supplementary Table S10). At the order level, *Anaerolineales* (*Anaerolineae* class, Supplementary Fig. S3), *Lactobacillales* (*Bacilli* class), *Clostridiales* (*Clostridia* class), *Rhodocyclales* (*Betaproteobacteria* class) and *Desulfobacterales* (*Deltaproteobacteria* class) showed a significant reduction in relative abundance in post-WWTP samples (Supplementary Fig. S4, Supplementary Table S10). In contrast, *Burkholderiales* (*Betaproteobacteria* class) and *Xanthomonadales* (*Gammaproteobacteria* class) significantly increased in relative abundance over time in post-WWTP samples (Supplementary Table S10).

### Bacterial diversity and richness indices

**Figure 4:**
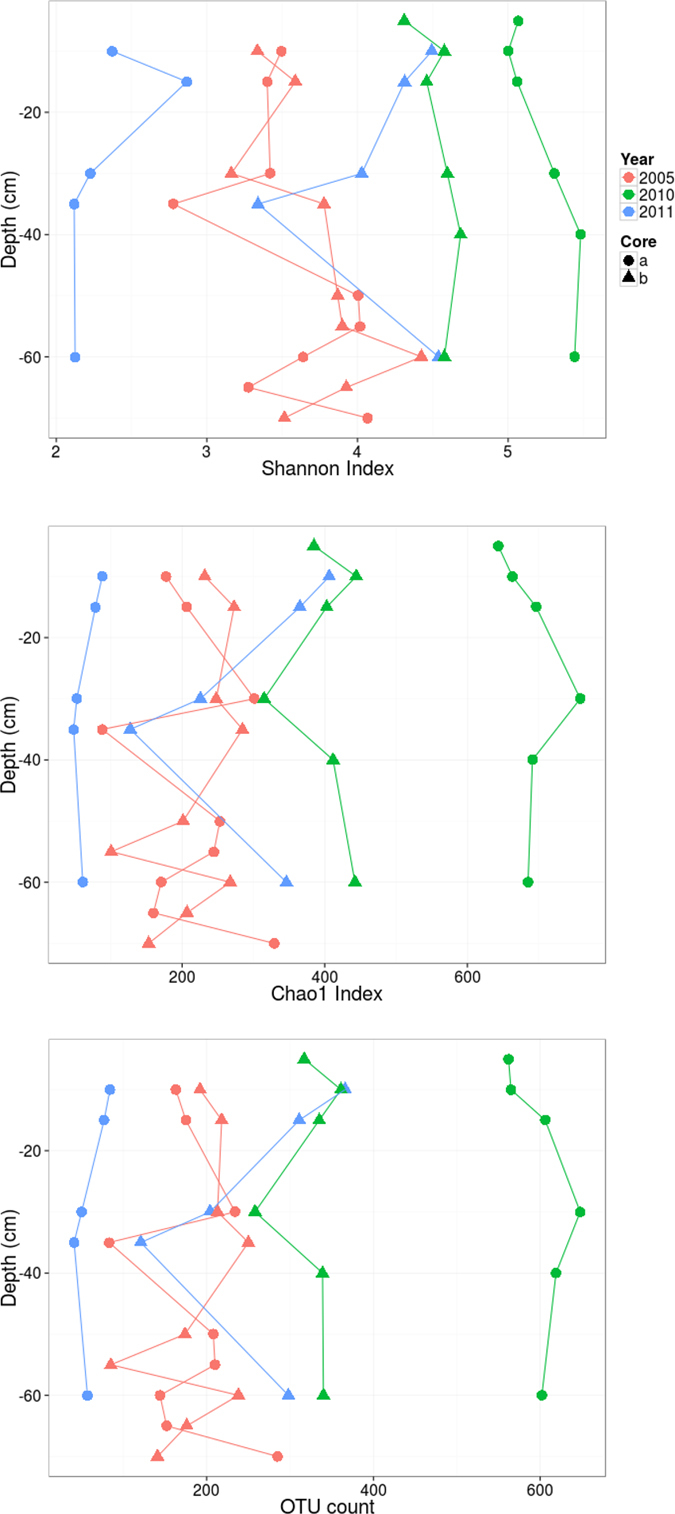
Diversity indices. Alpha diversity (Shannon) and richness (Chao1 and OTU count) indices at different depths in the sediment core samples.

In the sediment cores sampled in 2005, the alpha diversity (Shannon) and richness (Chao1 and OTU count) indices did not show significant difference between two cores (Fig. 4, Supplementary Table S11). Similarly, the alpha diversity and richness of the post-WWTP samples of core-B of 2010 and core-B of 2011 did not show significant differences with the pre-WWTP samples. However, the alpha diversity and richness indices were the highest and lowest in the core-A of 2010 and core-A of 2011, respectively (Fig. 4, Supplementary Table S11).

### Correlation between environmental parameters and microbial community composition

**Figure 5:**
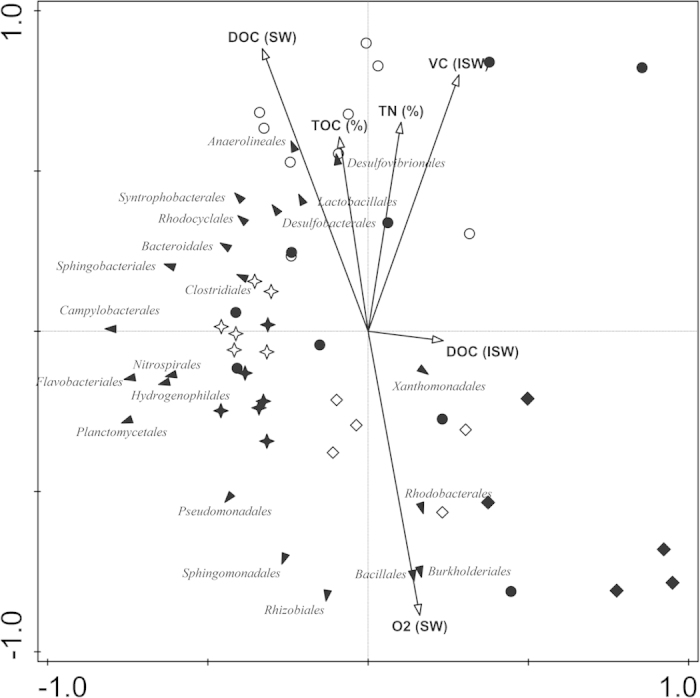
Redundancy analysis (RDA). Triplot of the relationship between microbial composition at order level and environmental parameters. Orders are indicated with filled triangles, environmental parameters with black arrows. The samples of 2005, 2010, and 2011 are shown as circle, star and diamonds, respectively, with core-a samples as filled and core-b samples as empty symbols. The eigenvalues of the first (*x*) and second (*y*) canonical axes are 0.262 and 0.194, respectively. The arrow length and direction corresponds to the variance that can be explained by the environmental variable. The direction of an arrow indicates an increasing magnitude of the environmental variable. The perpendicular distance between orders and environmental variable axes in the plot reflects their correlations. The smaller the distance, the stronger the correlation. SW: surface water; ISW: interstitial water.

We performed multivariate analysis (RDA) to study the relationship of environmental parameters and bacterial community composition at the order level (Fig. 5). The 2005 samples were mainly correlated with the organic carbon and nitrogen load, i.e. DOC content of surface water and TOC and TN contents in the sediment. The 2011 samples showed the opposite pattern while being positively correlated with the O_2_ concentration in surface water. The samples of 2010 were distinctly grouped in the centre and along the second canonical axis (i.e., the *y* axis in Fig. 5) with the lowest between-sample variation. In parallel, there was a clear trend on positioning of the bacterial orders in relation to environmental parameters. The bacterial orders *Xanthomonadales, Rhodobacterales, Burkholderiales, Bacillales, Rhizobiales, Sphingomonadales* and *Pseudomonadales* were positively correlated with the oxygen concentration in surface water while they were negatively correlated with DOC concentration in surface water and TOC and TN contents in the sediment. In contrast, *Desulfovibrionales*, *Anaerolineae*, *Lactobacillales*, *Desulfobacterales, Syntrophobacterales* and *Rhodocyclales* showed negative correlation to oxygen concentration in surface water while being positively correlated with DOC content of surface water and TOC content of the sediment (Fig. 5).

## Discussion

The objective of this study was to assess the long-term impact of the implementation of a WWTP on the size, structure and function of microbial communities residing in the hyporheic zone of a stretch of the Belgian Zenne River impacted by a VC-contaminated groundwater plume. Previous studies on riverbed sediments impacted by WWTP effluent showed decreased microbial diversity^26^,^27^ and an altered nitrogen cycle^28^ in the receiving rivers. However, the potential impact of the WWTP effluent on the downstream river can be influenced by a number of criteria, in particular the applied treatment technology that has been shifting towards advanced tertiary treatment in European countries in recent years.

Decades of municipal sewage input to the Zenne River at various locations in the vicinity of the test area caused high enrichment of TOC and TN in the pre-WWTP sediments. In turn, the establishment of the WWTP led to significant DOC decrease and O_2_ increase in the Zenne surface water. This translated into effective reduction of TOC and TN in post-WWTP sediments in particular in the surficial sediment horizons (Fig. 1a, Supplementary Table S4–6). Concomitant with this transition from nutrient-rich to nutrient-poor conditions, we observed a remarkable reduction in abundance of phylogenetic and functional biomarkers. qPCR analysis showed the highest copy numbers of bacterial 16S rRNA genes and genetic biomarkers associated with sulphate reducers (*dsrB*), denitrifiers (*nirS, nirK, nosZ*) and methanogens (*mcrA*) in pre-WWTP sediments that in most cases significantly decreased in post-WWTP sediments (Supplementary Table S6). Depletion of labile organic carbon and nitrogen sources and establishment of more oxic conditions in the post-WWTP sediment could be the driver of this pattern as especially sulphate reducers and methanogens show highest activity under nutrient-rich and highly reducing conditions^30^. The higher abundance of ammonium-oxidising bacteria and archaea (*amoA*) in pre-WWTP sediments could be due to ample presence of nitrogen resources (Fig. 1a).

Consistent with this trend, we observed reduced relative abundance of bacterial guilds that are known for their heterotrophic, syntrophic and fermentative lifestyle. On the other hand, the nutrient-poor conditions following the construction of the WWTP in our study likely favored members of the community that have higher substrate affinity and are more competitive under low nutrient conditions characterized by the presence of more recalcitrant complex organic substrates. For example, the pre-WWTP sediment and in particular the nutrient-rich shallow layers contained high relative abundance of *Chloroflexi* and in particular members of the order *Anaerolineales* that are known heterotrophs capable of metabolizing carbohydrates and peptides^31^. We noticed a strong correlation between *Anaerolineales* and DOC content in surface water and the sediment TOC and TN content (Fig. 5). The decreased relative abundance of *Chloroflexi* in the riverbed sediment downstream of a WWTP was reported previously where a lower organic carbon load was noted^26^. Moreover, a recent community genomics study expanded the potential contribution of *Chloroflexi* in sediment carbon cycling to respiration of sugars, fermentation, and acetogenesis^32^. Besides *Anaerolineales*, the high loads of organic carbon coupled to high microbial activity (and hence subsequent oxygen depletion) in the pre-WWTP hyporheic sediments favored other bacterial community members that are active under anoxic and sub-oxic conditions. Accordingly, bacterial fermenters, such as members of the *Lactobacillales* and *Clostridiales*, sulphate-reducing *Desulfobacterales* and denitrifying members of *Rhodocyclales* showed strong positive correlations with organic carbon content of the surface water and sediment (Fig. 5). Besides, these microbial guilds had the highest relative abundances in pre-WWTP sediments (Supplementary Fig. S4) and significantly reduced in post-WWTP samples (Supplementary Table S10) that is consistent with the quantified biomarkers (for SRBs and denitrifiers, Supplementary Fig. S4).

The low-organic carbon concentrations after the installation of the WWTP also correlated with reduced abundance of *D. mccartyi*, which belongs to the *Chloroflexi* and is currently the only OHRB known to completely dechlorinate CAHs to ethene. Similarly, non-dechlorinating anaerobes (e.g. SRBs, methanogens, fermenters) that are commonly found to coexist with OHRB, and have been suggested to drive OHR by syntrophic interactions and exchange of essential metabolites^32^,^33^,^34^, were reduced in relative abundance in post-WWTP samples^33^,^34^,^35^. However, this did not impact VC dechlorination potential in the microcosms prepared from the sediments of 2010 as compared with the microcosms of 2005. Although diminished but consistent VC dechlorination was noted for the 2011 sediments, subsequent stimulation of VC dechlorination by tree bark addition showed that the OHRB and supporting non-dechlorinators were still present, albeit at lower initial abundance, and could be stimulated by re-establishment of organic-rich conditions.

In contrast, the reduced nutrient availability in post-WWTP sediments coincided with enrichment of certain proteobacterial orders. For instance, the proportion of *Burkholderiales* sequences showed a significant increase over time especially in the deeper sediment horizons (Supplementary Fig. S4) with a positive correlation to nutrient-poor and oxic conditions (Fig. 5). Members of the *Burkholderiales* have versatile catabolic traits enabling them to degrade recalcitrant and aromatic compounds and survive in environments with limited nutrient availability^36^,^37^. Bacterial orders of *Xanthomonadales*, *Pseudomonadales*, and *Sphingomonadales* that are known to be active under oxic conditions and can survive nutrient-poor conditions were positively correlated with oxygen concentration in surface water, and showed a negative correlation to organic carbon content in surface water and sediment (Fig. 5). The wide metabolic flexibility and possession of high affinity transporters for substrates and nutrients makes *Pseudomonadales* capable of thriving in oligotrophic environments^38^. Similarly *Xanthomonadales* members were proposed to survive in niches where nutrients are limited by decomposing recalcitrant carbon sources such as hemicellulose^39^ and members of the *Sphingomonadales* were considered to be adapted to conditions of low availability of metabolizable substrates^40^.

When we compared the bacteria alpha diversity (Shannon) and richness (Chao1, OTU count) between the pre- and post-WWTP sediment samples, one of the 2010 cores showed significantly higher values compared to the remaining cores, whereas one of the cores of 2011 had significantly lower richness and diversity. Conflicting results have been reported for the impact of WWTP on sediment bacterial diversity with some showing an increase in bacterial diversity downstream of a WWTP effluent input^28^,^41^ while others found decreased diversity^26^,^27^. It should be noted that we cannot exclude the impact of other parameters such as season-dependent community dynamics in our study as the three sampling campaigns were performed in three different seasons (winter 2005, summer 2010, and fall 2011). Sediment microbial communities have been shown to be spatially heterogeneous and correlated with seasonal changes in physicochemical parameters such as temperature^15^,^16^,^21^,^25^, DOC content^15^,^16^,^25^, nitrate content^15^,^23^, and phosphate concentration^15^. Moreover, the decreased concentrations of CAHs reaching the river could also impact the community members such as OHRB.

Our study showed that operation of a technologically advanced WWTP effectively promoted low-nutrient conditions in the longer term. This was translated into transition of the bacterial community in the riverbed hyporheic sediment from members favoured under nutrient-rich and reducing conditions to those adapted to nutrient-poor and oxic conditions, indicating that microbial communities are good sentinels and integrators of environmental change. The low-organic condition did not have a detrimental impact on VC respiration as one of the important hyporheic functions at CAH-contaminated sites fuelled by organic matter. Overall, the results of this study showed a positive impact of the technologically advanced WWTP on the sediment bacterial community, in line with sustainable water resource management aims.

## Methods

### Site description

**Figure 6:**
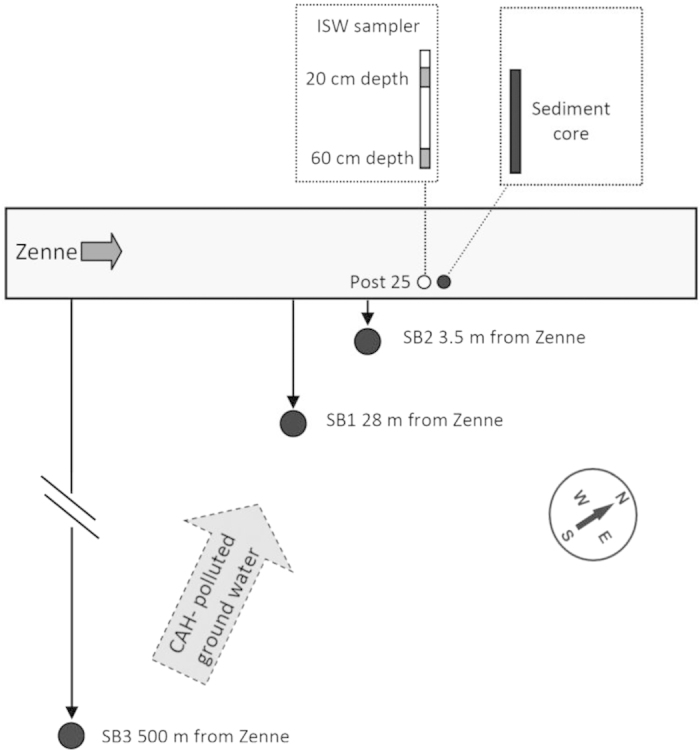
Schematic representation of the test site. The locations of interstitial water samplers (ISW) and sediment core sampling at post 25 and of groundwater monitoring wells (large black dots) monitored in this study. Flow directions of the CAH plume and Zenne River are indicated by arrows.

The study site is a short transect of the Zenne River located in an industrial area near Brussels, Belgium. The test site was previously described in detail^12^, and a simplified schematic presentation is given in Fig. 6 Briefly, at the study site, the Zenne River is relatively straight, 12–15 m wide, 0.5–2 m deep with a stream flow of 5–10 m^3^/s in dry weather conditions^13^. Based on data from October 2004 until January 2007, the nearby aquifer was shown to be contaminated with tetrachloroethene (PCE), trichloroethene (TCE) and 1,1,1-trichloroethane, originating from several sources^12^. Moreover, it was noted that *cis*-dichloroethene and in particular VC as degradation products of reductive dechlorination of PCE and TCE, were reaching to the Zenne riverbed sediment^12^,^29^. Since 2009 the concentration of CAHs that reach the river has decreased while the concentration of contaminants remained stable in the source area. It is unclear, however, whether this is due to increased OHR activity during plume flow from the source towards the river, or rather due to plume diversion.

### Sample collection

Sediment cores were collected from the top 80-cm layer of the riverbed sediment in December 2005, May 2010 and September 2011, at post 25 (P25, Fig. 6) using a 4-cm-diameter piston sediment sampler. At all three time-points, triplicate sediment cores were collected at a distance of approximately 50 cm from each other, randomly designated as cores A, B, or C. Two of the sediment cores (A and B) were preserved immediately on dry ice to conserve the spatial structure of the sediment layers. These cores were kept at −20 °C until DNA extraction in November 2011 as described below. Core C was transferred directly to the lab in a cooler and used to set up microcosms to study OHR potential. Groundwater, surface water, sediment pore water sampling, as well as preservation were performed as reported previously^13^. Briefly, groundwater samples were collected from the monitoring well SB-1 (Fig. 6), and surface water samples were collected from the river as grab samples in glass bottles, without leaving a headspace. Electrical conductivity, dissolved oxygen and pH were measured regularly (Table 1) in the surface water using an electrometric multimeter (MultiLine F/SET3, WTW, Weilheim, Germany) and appropriate electrodes (TetraCon 325, CellOx 325 and Sen Tix 41 from WTW, Weilheim, Germany, respectively). Sediment interstitial water (ISW) was collected from Teflon pore water samplers that were permanently installed in the test area near the right riverbank at two different depths in the river sediment (20 and 60 cm) as reported previously^13^.

### Microcosm set up

Riverbed sediment microcosms were prepared directly after each sampling campaign with samples taken from the top (0–20 cm depth) sediment layer of the core C samples. The microcosms were prepared in an anoxic glove box (Don Whitley Scientific ltd, West Yorkshire, UK) containing an atmosphere of N_2_. Approximately 37 g (wet) sediment was transferred to 160-ml glass serum bottles containing 70 ml groundwater from the monitoring well SB-1. All microcosms were prepared in duplicate. Two extra microcosms prepared from the core C sample of 2011 were supplemented with 1 g tree bark as a source of solid organic carbon and electron donor as described elsewhere^42^. All microcosms were spiked with VC giving a final aqueous concentration of 2 mg/L. The microcosms containing sediment from 2005 were first allowed to degrade residual VC that was present in the groundwater, before VC was added.

### DNA extraction, PCR-DGGE and qPCR

All A and B sediment cores were divided into 1-cm slices until a depth of 60–70 cm. At 5 cm intervals, a total of 80 sediment samples of approximately 2 g were used for DNA extraction as described previously^43^. DGGE fingerprinting of the bacterial 16S rRNA gene pool in these 80 samples was performed as described previously^44^, and 40 samples were chosen for further molecular analysis. The 16S rRNA gene of bacteria and *D. mccartyi* and selected protein-encoding genes commonly used as biomarkers for specific functional guilds, i.e. sulphate reducers (*dsrB*), denitrifiers (*nirK*, *nirS*, *nosZ*), methanogens (*mcrA*), and bacterial/archaeal ammonium oxidizers (bacterial/archaeal *amoA*) were quantified for the 40 selected samples. The qPCR measurements were performed in triplicate in 25-μL reactions in an iQ5 iCycler (Bio-Rad, Veenendaal, the Netherlands) using the iQ SYBR Green Supermix kit (Bio-Rad). See Supplementary Table S1 for the lists of the primers and thermal cycling conditions used for qPCRs in this study. Standard curves were obtained using serial dilutions of a known amount of plasmid DNA containing a fragment of the respective genes. Genomic DNA from *Methanosarcina mazei* (ATCC BAA-159; GenBank accession number NC_003901) was used as standard for *mcrA* quantification.

### Bacterial 16S rRNA gene amplicon pyrosequencing

Bacterial 16S rRNA genes were first amplified from the DNA extracts using the general 27F/1492R primer set^45^ (Supplementary Table S2). In a second PCR barcoded amplicons covering the V1-V2 region of the bacterial 16S rRNA gene were generated using the 27F-DegS primer as the forward primer^46^ and an equimolar mix of the two reverse primers, 338R I and II^47^. The PCR mix (100 μl final volume) contained 20 μl of 5 × HF buffer (Finnzymes, Vantaa, Finland), 2 μl PCR Grade Nucleotide Mix (Roche Diagnostic GmbH, Mannheim, Germany), 1 μl of Phusion Hot start II High-Fidelity DNA polymerase (2 U/μl) (Finnzymes), 500 nM of the reverse primer mix and the forward primer (Biolegio BV, Nijmegen, The Netherlands), 2 μl (i.e. 40 ng) template, and 65 μl nuclease free water. PCR was performed using the following conditions: 98 °C for 30 s, followed by 30 cycles consisting of denaturation at 98 °C for 10 s, annealing at 56 °C for 20 s, elongation at 72 °C for 20 s, and a final extension at 72 °C for 10 min. The PCR product was quality-checked on a 1% agarose gel, purified from gel using the High Pure PCR Cleanup Micro Kit (Roche Diagnostics) and its concentration was determined by a Qubit 2.0 Fluorometer (Life Technologies, Darmstadt, Germany). Amplicons were sequenced using an FLX genome sequencer in combination with titanium chemistry (GATC-Biotech, Konstanz, Germany).

### Analysis of the pyrosequencing data

Pyrosequencing data were processed and analysed using QIIME v1.8.0^48^. Raw sequencing data was first pre-processed according to default options in QIIME to remove low quality and ambiguous reads. Pre-processed data was denoised^49^ to minimize sequencing errors in the dataset and chimeric sequences were removed using UCHIME^50^. Operational taxonomic units (OTUs) were defined at a 97% identity level using uclust^51^ and the SILVA 111 database^52^ as a reference. The full pre-processed data was transformed into relative abundances before comparing taxon abundances between samples. Note that the relative abundance comparisons between the different taxa are not independent, and may be therefore affected by the compositionality effect^53^. Bacterial diversity and richness indices were determined with R (3.1.2)^54^ using the phyloseq package (1.9.15)^55^ form the complete dataset without rarification^56^.

### Analytical methods

Concentrations of CAHs, ethene, and ethane were determined via head-space analysis on a Varian GC-FID (CP-3800) as described previously^57^. Total organic carbon (TOC) and total nitrogen (TN) were measured using the oxidative digestion method (C/N analyzer EA1110). Concentrations of sulphate, nitrate, phosphate, and chloride were analyzed by ion chromatography using a Dionex DX-120 ion chromatograph equipped with a Dionex AS14A column (Dionex, Sunnyvale, CA). Dissolved organic carbon (DOC) was determined from samples as the difference between total dissolved carbon and dissolved inorganic carbon, measured with a Shimadzu TOC-5000 analyzer equipped with an ASI-5000 auto-sampler.

### Statistical analyses

Univariate statistical analysis (Spearman correlation and Kruskal-Wallis test) were performed within the Matlab environment (Version 2015a, The Mathworks, Natick, MA), unless otherwise stated. For all statistical analysis, Benjamini-Hochberg^58^ correction for multiple testing was applied for false discovery rate (FDR)-controlling; a q-value < 0.25 was deemed significant^59^,^60^. The phylogenetic profiling data was analysed with R (3.1.2)^54^ using the phyloseq package (1.9.15)^55^. We included OTUs that were present in at least three samples and contained one or more reads. This resulted in a final set of 1303 detected OTUs (out of 21067 total OTUs). In order to relate the changes in microbial communities (at order level) with environmental variables, redundancy analysis (RDA) was used as implemented in the CANOCO 5 software (Biometris, Wageningen, The Netherlands). The environmental variables tested were DOC and oxygen in surface water, TOC and TN in the sediment cores, and DOC and VC concentration in ISW. Nitrite and nitrate in ISW were not considered as their levels were always below detection limit. The community structure was visualized via ordination triplots with scaling focused on intersample differences.

## Additional Information

**Accession codes:** The complete dataset is available at the European Bioinformatics Institute (www.ebi.ac.uk) under accession number PRJEB8703.

**How to cite this article**: Atashgahi, S. *et al.* Impact of a wastewater treatment plant on microbial community composition and function in a hyporheic zone of a eutrophic river. *Sci. Rep.* **5**, 17284; doi: 10.1038/srep17284 (2015).

## Supplementary Material

Supplementary Information

## Figures and Tables

**Table 1:** Physico-chemical parameters of surface water.

Date	Concentration (mg/L)									DOC (mg/L)	O_2_(mg/L)	Temp (°C)	pH	Conductivity (μS/cm)
	PO_4_	SO_4_	NO_2_	NO_3_	Cl	Fe	Na	Mg	K					
**Dec 2005**	**—**a	**—**	**—**	**—**	**—**	**—**	**—**	**—**	**—**	**32**	**1.2**	**—**	**—**	**—**
May 2006	2.32	214	BD^b^	0.68	258	0.51	90	15	18	70	**—**	17.8	7.88	1126
Dec 2006	**—**	**—**	**—**	**—**	**—**	**—**	**—**	**—**	**—**	**—**	1.9	14.5	7.62	1576
***Mar 2007***	***0.67***	***103***	***BD***	***7.52***	***95***	**—**	**—**	**—**	**—**	**—**	***3.6***	***12.9***	***7.62***	***1542***
May 2007	1.97	58	BD	BD	148	0.58	80.3	14.9	16.2	**—**	3.7	13.2	7.58	1368
Nov 2007	0.82	101	BD	6.70	79	0.09	44.7	13.7	9.6	**—**	**—**	7.5	8.03	791
Oct 2008	**—**	73	BD	BD	61	0.11	**—**	**—**	**—**	**—**	5.9	15.7	8.46	952
Feb 2010	**—**	65	**—**	13.9	92	0.08	63	11	10	15.85	5.5	11.5	7.32	911
**May 2010**	**—**	**111**	**0.56**	**10.4**	**104**	**0.02**	**81**	**17**	**13**	**19.73**	**4.2**	**13.9**	**7.47**	**1122**
Sep 2010	1.80	102	BD	6.14	53	0.01	71	14	15	13.12	4.4	18.2	7.42	962
Jan 2011	**—**	**—**	**—**	**—**	**—**	**—**	**—**	**—**	**—**	**—**	6.5	7.4	7.42	1070
May 2011	**—**	100	**—**	**—**	140	0.17	**—**	**—**	**—**	6.55	4.7	18	7.38	1211
**Sep 2011**	**1.10**	**78**	**0.15**	**2.4**	**79**	**0.01**	**75**	**12**	**9.7**	**7.86**	**5.3**	**17.1**	**7.47**	**777**

The dates of sediment core samplings are shown in boldface and the date of WWTP installation (Mar 2007) is shown in bold/italic.

^a^No data.

^b^Below detection limit.

**Table 2:** Physico-chemical parameters of interstitial water sampled from the permanent pore water samplers in the riverbed.

Date	Sampling depth (cm)	Concentration (mg/L)				
		DOC	SO_4_	NO_2_	NO_3_	VC
Dec 2005	20	5	170	BD^a^	BD	0.02
	60	4	198	BD	BD	1.2
May 2010	20	3.7	145	BD	BD	0
	60	3.5	144	BD	BD	0
Sep 2011	20	6.3	140	BD	BD	0
	60	2.6	140	BD	BD	0

^a^BD: Below the detection limit.
