# Supplementary material for: Impact of a wastewater treatment plant on microbial community composition and function in a hyporheic zone of a eutrophic river
Source: Sci Rep. 2015 Nov 26;5:17284. doi: 10.1038/srep17284 (PMC4660315; doi:10.1038/srep17284)
Supplement: Supplementary Information [file srep17284-s1.pdf]

Supplementary Information

Impact of a wastewater treatment plant on microbial community composition and function in a hyporheic zone of a eutrophic river

Siavash Atashgahi, Rozelin Aydin, Mauricio R. Dimitrov, Detmer Sipkema, Kelly Hamonts, Leo Lahti, Farai Maphosa, Thomas Kruse, Edoardo Saccenti, Dirk Springael, Winnie Dejonghe & Hauke Smidt

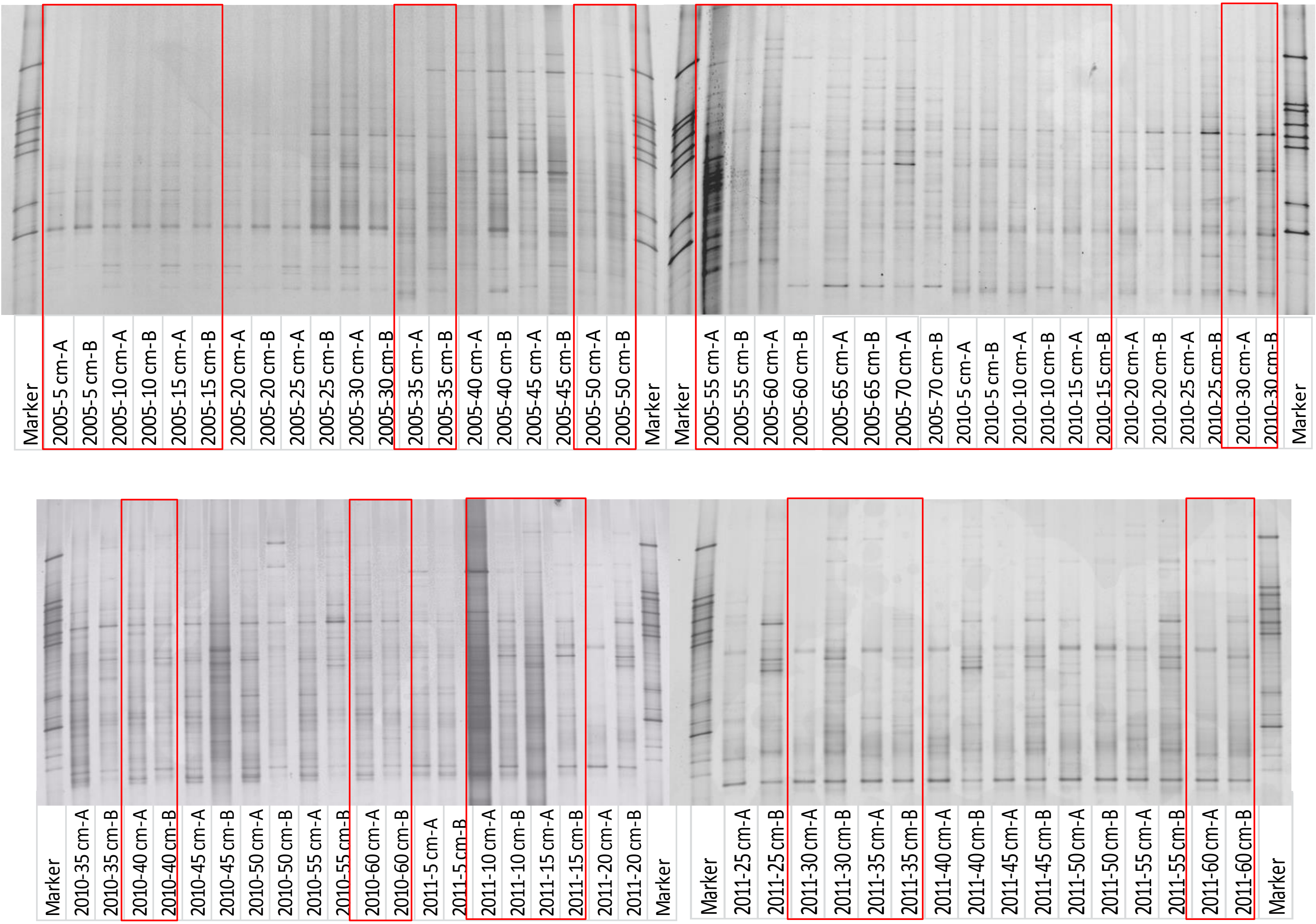

Figure S1. DGGE profiles obtained from each 5 cm interval of the duplicate sediment cores from 2005, 2010 and 2011. The samples used for further molecular analysis are shown in red boxes.

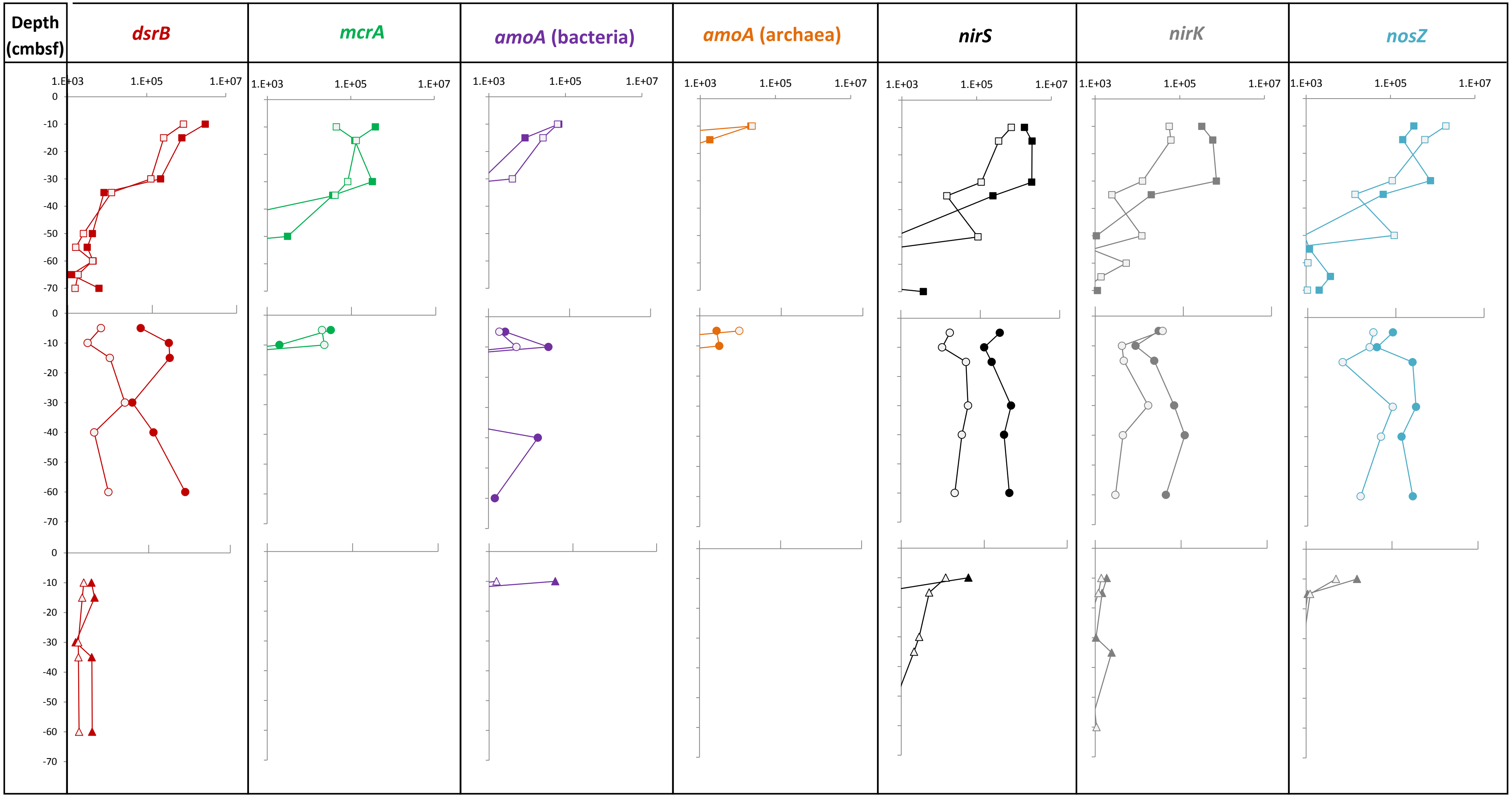

Figure S2. Copy numbers of sulphate reducers (*dsrB*) and methanogens (*mcrA*), *amoA* (bacteria), *amoA* (archaea), *nirS*, *nirK*, and *nosZ* genes at different depths in the duplicate sediment core samples taken in 2005, 2010 and 2011, as determined by qPCR. Each value represents the average value obtained from triplicate qPCRs performed on one sample.

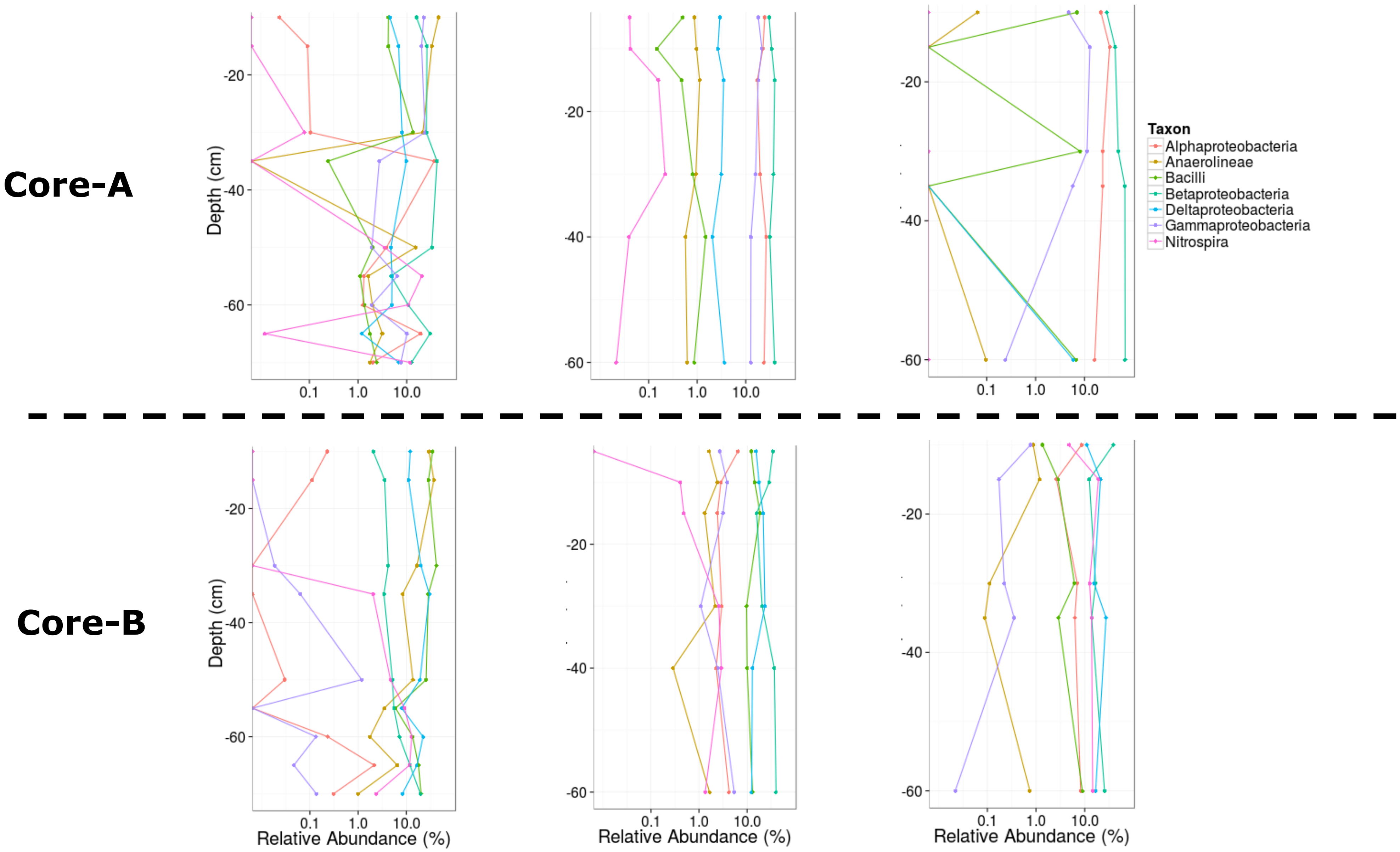

Figure S3. Log-scale relative of the dominant bacterial classes at different depths in the duplicate sediment core samples taken in 2005 (right), 2010 (middle) and 2011 (left).

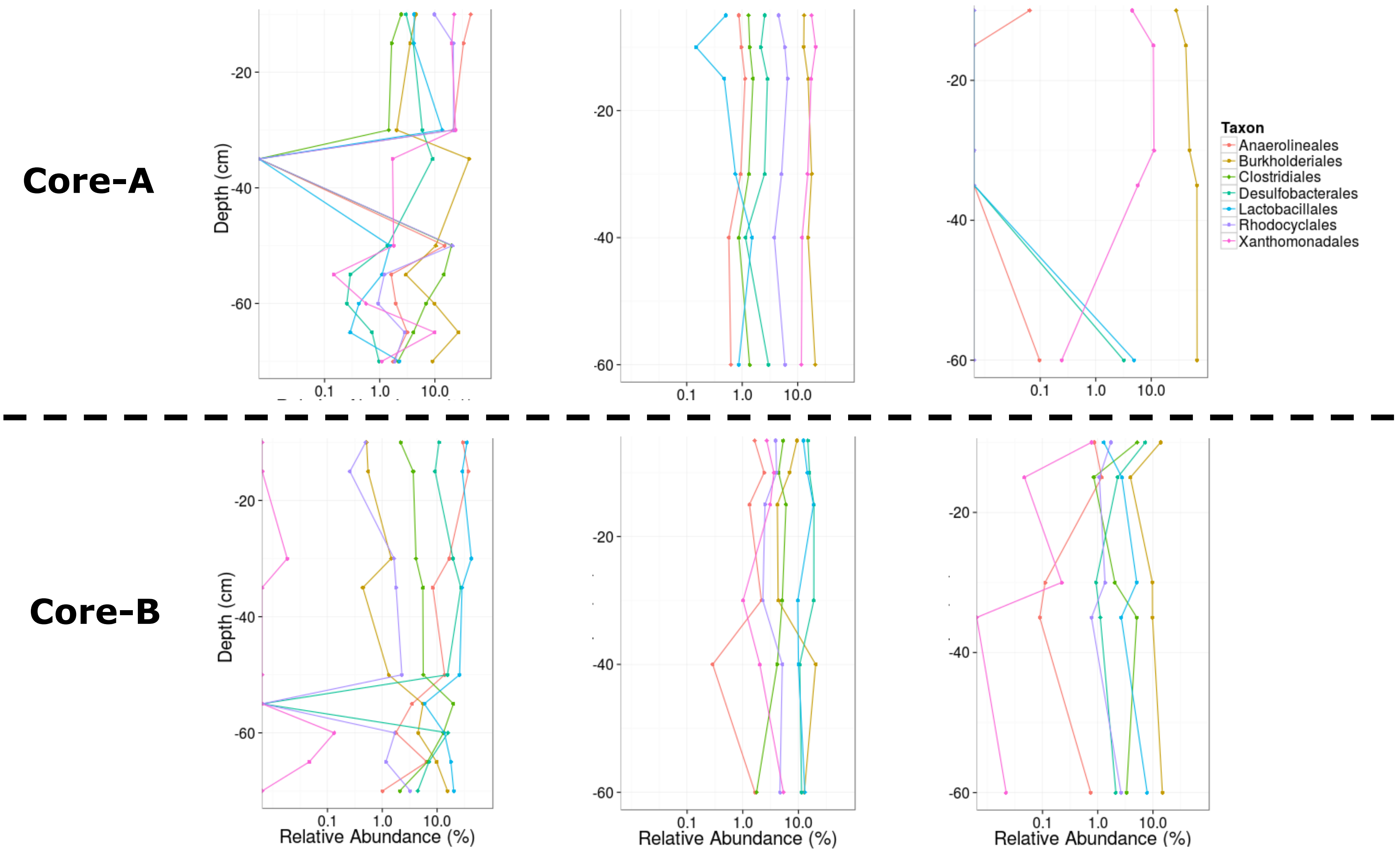

Figure S4. Log-scale relative of the dominant bacterial orders at different depths in the duplicate sediment core samples taken in 2005 (right), 2010 (middle) and 2011 (left).

Table S1. Overview of primer sequences and thermal cycling conditions used for qPCR in this study.

Primer	Oligonucleotide sequence (5´-3´)	Thermal profile	Number of cycles	Target	Reference
341F	CCTACGGGAGGCAGCAG	95 °C 10 min	1	Bacteria	[1]
534R	ATTACCGCGGCTGCTGGC	95 °C 15 s, 60 °C 30 s, 72 °C 30 s	40		
Dco728F	AAGGCGGTTTTCTAGGTTGTCAC	95 °C 10 min	1	*D. mccartyi*	[2]
Dco944R	CTTCATGCATGTCAAAT	95 °C 15 s, 50 °C 30 s, 72 °C 30 s	40		
dsrP2060F	CAACATCGTYCAYACCCAGGG	95 °C 15 min	1	*dsrB*	[3]
DSR4R	GTATAGCAGTTACCGCA	95 °C 30 s, 55 °C 45 s, 72 °C 30 s	40		
ME1 fwd	GCMATGCARATHGGWATGTC	95 °C 15 min	1	*mcrA*	[4]
ME3 rev	TGTGTGAAWCCKACDCCACC	94 °C 10 s, 54 °C 20 s, 72 °C 30 s	40		
Bact amoA-1F	GGGGTTTCTACTGGTGGT	50 °C 2 min, 95 °C 10 min	1	*amoA* (bacteria)	[5]
Bact amoA-2R	CCCCTCKGSAAAGCCTTCTTC	95 °C 30 s, 58 °C 40 s, 72 °C 1 min	45		
Arch amoAF	STAATGGTCTGGCTTAGACG	50 °C 2 min, 95 °C 10 min	1	*amoA* (archaea)	[5]
Arch amoAR	GCGGCCATCCATCTGTATGT	95 °C 30 s, 56 °C 40 s, 72 °C 1 min	45		
nirSCd3aF	AACGYSAAGGARACSGG	95 °C 10 min	1	*nirS*	[6]
nirSR3cd	GASTTCGGRTGSGTCTTSAYGAA	95°C 15 s, 56°C 30 s, 72°C 30 s	40		[7]
nirK876	ATYGGCGGVCAYGGCGA	95 °C 10 min	1	*nirK*	[8]
nirK1040	GCCTCGATCAGRTTRTGGTT	95°C 15 s, 60°C 30 s, 72°C 30 s	40		
nosZ2F	CGCRACGGCAASAAGGTSMSSGT	95 °C 10 min	1	*nosZ*	[9]
nosZ2R	CAKRTGCAKSGCRTGGCAGAA	95°C 30 s, 60°C 30 s, 72°C 30 s	40		

Table S2. Oligonucleotide primers and sequence adapters used for pyrosequencing in this study.

Primer[a]	Oligonucleotide sequence (5´-3´)[b]	Reference
Adaptor A	CCATCTCATCCCTGCGTGTCTCCGACTCAG	Provided by GATC-Biotech
Adaptor B	CCTATCCCCTGTGTGCCTTGGCAGTCTCAG	Provided by GATC-Biotech
27F-DegS	GTTYGATYMTGGCTCAG	[10]
338R-I	GCWGCCTCCCGTAGGAGT	[11]
338R-II	GCWGCCACCCGTAGGTGT	[11]
27F	AGAGTTTGATCCTGGCTCAG	[12]
1492R	GGTTACCTTGTTACGACTT	[12]

[a] Primer names may not correspond to original publication
[b] M = A or C; R = A or G; W = A or T; Y = C or T

Table S3. Spearman correlation (ρ) and associated q-values of environmental parameters in surface water during the experimental period and Kruskal-Wallis test q-values for comparison between the pre- and post-WWTP parameters.

Variable	ρ	q-value	Kruskal-Wallis q-value
PO_4	-0.257	0.768	1.077
SO_4	-0.224	0.768	0.237
NO_2	-1.000	1.000	NA [a]
NO_3	0.036	1.000	0.558
Cl	-0.432	0.497	0.333
Fe	-0.703	0.142	0.378
Na	-0.286	0.768	0.312
Mg	-0.357	0.768	0.444
K	-0.500	0.533	0.312
DOC	-0.893	0.090	0.237
O_2	0.709	0.090	0.200
Temp	0.161	0.768	0.912
pH	-0.661	0.090	0.327
Cond	-0.524	0.235	0.234

[a] NA: not applicable

Table S4. Characteristics of duplicate sediment cores taken in 2005, 2010 and 2011 including total organic carbon (TOC) and total nitrogen (TN) content, 16S rRNA gene copy numbers of bacteria and *D. mccartyi* (as measured by qPCR). Each qPCR value represents the average and standard deviation obtained from triplicate reactions.

Core samples	TOC (%)		TN (%)		Bacteria				*D. mccartyi*			
	Core-A	Core-B	Core-A	Core-B	Core-A		Core-B		Core-A		Core-B	
					Copies/g	SD [a]	Copies/g	SD	Copies/g	SD	Copies/g	SD
2005.10cm	1.569	2.274	0.043	0.074	1.3E+08	1.2E+07	3.3E+07	4.3E+06	1.2E+03	4.1E+02	7.2E+04	1.9E+04
2005.15cm	0.852	2.904	0.025	0.086	1.7E+08	4.4E+06	9.3E+06	2.1E+05	2.6E+03	6.9E+02	2.1E+04	6.2E+03
2005.30cm	0.210	0.479	0.000	0.000	1.1E+07	8.8E+06	2.6E+06	4.5E+05	2.6E+04	3.0E+03	7.1E+03	1.2E+03
2005.35cm	0.968	0.453	0.045	0.014	6.3E+05	3.8E+05	3.4E+05	1.7E+04	1.8E+02	7.0E+01	7.7E+02	5.6E+02
2005.50cm	0.655	0.318	0.000	0.010	1.6E+05	1.4E+05	2.8E+04	4.6E+03	0	0	7.6E+02	4.4E+01
2005.55cm	0.108	0.153	0.006	0.000	1.9E+05	1.8E+05	1.1E+04	8.7E+02	2.4E+02	5.2E+01	0	0
2005.60cm	0.132	0.114	0.000	0.000	1.2E+05	1.2E+05	4.1E+04	1.4E+04	0	0	8.3E+02	6.1E+02
2005.65cm	0.198	0.198	0.000	0.006	3.8E+05	5.4E+04	3.9E+04	8.1E+03	0	0	0	0
2005.70cm	0.212	0.099	0.002	0.000	6.4E+05	7.4E+05	2.8E+04	4.3E+03	5.9E+02	3.7E+02	0	0
2010.5cm	0.426	0.429	0.027	0.000	4.8E+06	1.5E+06	8.4E+05	2.5E+05	7.0E+02	3.0E+02	7.8E+02	0
2010.10cm	0.382	0.296	0.014	0.007	3.7E+07	2.4E+06	2.0E+05	6.8E+03	2.6E+03	3.2E+02	0	0
2010.15cm	0.118	0.394	0.004	0.000	4.8E+05	5.5E+04	7.6E+05	2.3E+05	0	0	2.0E+02	0
2010.30cm	0.067	0.229	0.006	0.000	4.5E+05	3.0E+04	1.0E+06	8.7E+05	1.0E+02	8.1E+01	2.6E+02	0
2010.40cm	0.122	0.204	0.000	0.007	5.6E+06	4.7E+06	7.1E+05	4.9E+05	4.0E+02	4.3E+02	2.0E+02	0
2010.60cm	0.140	0.109	0.002	0.000	5.5E+07	9.3E+06	4.0E+05	8.4E+04	5.5E+02	2.2E+02	5.9E+02	4.7E+02
2011.10cm	0.162	0.196	0.006	0.000	5.0E+04	2.6E+03	2.1E+05	1.9E+05	0	0	0	0
2011.15cm	0.054	0.285	0.002	0.007	1.2E+05	1.1E+05	1.3E+05	1.2E+04	3.1E+02	2.5E+01	1.9E+02	0
2011.30cm	0.132	0.282	0.000	0.000	7.3E+04	2.2E+04	8.5E+04	3.2E+04	0	0	0	0
2011.35cm	0.089	0.244	0.004	0.000	6.8E+03	2.0E+03	4.2E+04	3.8E+04	0	0	0	0
2011.60cm	0.048	0.283	0.002	0.009	2.1E+04	4.9E+03	4.0E+04	2.5E+04	0	0	0	0

[a] SD: Standard deviation

Table S5. Spearman correlation (*ρ*) and associated q-values of evolution of TOC, TN, and 16S rRNA gene copy numbers of bacteria and *D. mccartyi* over sediment depth.

Cores	TOC		TN		Bacteria		*D. mccartyi*	
	ρ	q-value	ρ	q-value	ρ	q-value	ρ	q-value
2005-A	-0.690	0.185	-0.496	0.349	-0.617	0.245	-0.593	0.245
2010-A	-0.828	0.291	-0.886	0.185	0.314	0.796	-0.371	0.750
2011-A	-0.894	0.245	-0.200	0.873	-0.600	0.610	-0.354	0.873
2005-B	-0.937	0.015	-0.670	0.193	-0.717	0.185	-0.865	0.063
2010-B	-0.883	0.185	-0.101	0.904	0.486	0.610	0.029	0.983
2011-B	0.289	0.873	0.447	0.750	-0.200	0.873	-0.354	0.873

Table S6. Kruskal-Wallis test q-values for comparison between the pre- and post-WWTP parameters in sediment cores at top (0-20 cm) and bottom (20-70 cm) sediment horizons.

Variables	q-value sediment horizons	
	Top	Bottom
TOC	0.011	0.321
TN	0.011	1.000
Bacteria	0.011	0.067
D. mccartyi	0.011	1.000
dsrB	0.009	0.157
mcrA	0.009	0.208
amoA (bacteria)	0.030	0.384
amoA (archaea)	0.100	NA [a]
nirS	0.007	0.026
nirK	0.007	0.026
nosZ	0.007	0.026

[a] NA: not applicable

Table S7. Copy numbers of *dsrB* (sulphate reducers), *mcrA* (methanogens) and bacterial and archaeal *amoA* genes at different depths in the duplicate sediment core samples taken in 2005, 2010 and 2011, as determined by qPCR. Each value represents the average value obtained from triplicate qPCRs performed on one sample.

Core samples	*dsrB*				*mcrA*				*amoA* (bacteria)				*amoA* (archaea)			
	Core-A		Core-B		Core-A		Core-B		Core-A		Core-B		Core-A		Core-B	
	Copies/g	SD [a]	Copies/g	SD	Copies/g	SD	Copies/g	SD	Copies/g	SD	Copies/g	SD	Copies/g	SD	Copies/g	SD
2005.10cm	3.0E+06	6.8E+05	8.5E+05	2.9E+05	3.9E+05	1.8E+04	4.5E+04	3.1E+03	6.9E+03	2.8E+03	2.6E+03	1.2E+02	2.3E+03	1.0E+02	4.0E+03	1.5E+03
2005.15cm	6.8E+06	6.7E+05	2.7E+05	1.1E+05	1.3E+05	2.8E+04	1.4E+05	2.7E+04	8.8E+03	1.7E+03	2.6E+04	3.0E+03	1.8E+03	6.3E+02	0	0
2005.30cm	2.2E+05	9.0E+04	1.3E+05	1.9E+04	3.3E+05	5.3E+04	8.5E+04	3.2E+04	6.9E+02	3.6E+02	4.1E+03	2.0E+02	0	0	0	0
2005.35cm	8.3E+03	1.4E+03	1.3E+04	2.9E+03	3.7E+04	4.6E+04	4.2E+04	3.8E+04	0	0	0	0	0	0	0	0
2005.50cm	4.2E+03	6.4E+02	2.5E+03	2.9E+03	3.1E+03	2.4E+03	0	0	0	0	0	0	0	0	0	0
2005.55cm	3.1E+03	1.6E+03	1.6E+03	3.1E+02	0	0	0	0	0	0	0	0	0	0	0	0
2005.60cm	4.4E+03	2.4E+03	4.3E+03	2.8E+03	0	0	0	0	0	0	0	0	0	0	0	0
2005.65cm	1.2E+03	1.1E+03	1.8E+03	7.2E+02	0	0	0	0	0	0	0	0	0	0	0	0
2005.70cm	6.2E+03	2.1E+03	1.5E+03	4.1E+02	0	0	0	0	0	0	0	0	0	0	0	0
2010.5cm	5.3E+04	3.8E+03	6.1E+03	4.5E+03	3.2E+04	3.6E+04	2.0E+04	1.2E+03	2.6E+03	1.2E+03	1.9E+03	1.1E+03	2.6E+03	1.2E+03	9.3E+03	2.8E+02
2010.10cm	2.5E+05	3.9E+05	2.9E+03	1.2E+03	2.0E+03	1.7E+03	2.3E+04	4.5E+03	3.0E+04	5.7E+03	4.9E+03	1.3E+03	3.0E+04	5.7E+03	0	0
2010.15cm	2.6E+05	4.5E+04	9.9E+03	1.3E+03	0	0	0	0	0	0	0	0	0	0	0	0
2010.30cm	3.4E+04	3.7E+03	2.3E+04	1.6E+04	0	0	0	0	0	0	0	0	0	0	0	0
2010.40cm	1.1E+05	1.1E+04	4.2E+03	1.4E+03	0	0	0	0	1.7E+04	2.3E+03	0	0	0	0	0	0
2010.60cm	6.0E+05	1.3E+05	9.1E+03	8.7E+03	0	0	0	0	1.4E+03	9.4E+02	0	0	0	0	0	0
2011.10cm	4.0E+03	1.9E+03	2.6E+03	5.4E+02	0	0	0	0	3.8E+04	2.7E+03	1.5E+03	1.0E+02	0	0	0	0
2011.15cm	4.7E+03	1.3E+03	2.4E+03	1.3E+03	0	0	0	0	0	0	0	0	0	0	0	0
2011.30cm	1.6E+03	7.0E+02	1.9E+03	3.1E+02	0	0	0	0	0	0	0	0	0	0	0	0
2011.35cm	4.0E+03	1.5E+03	1.9E+03	1.3E+03	0	0	0	0	0	0	0	0	0	0	0	0
2011.60cm	4.1E+03	9.7E+02	2.0E+03	1.1E+03	0	0	0	0	0	0	0	0	0	0	0	0

[a] SD: Standard deviation

Table S8. Copy numbers of *nirS*, *nirK* and *nosZ* (denitrifiers) at different depths in the duplicate sediment core samples taken in 2005, 2010 and 2011, as determined by qPCR. Each value represents the average value obtained from triplicate qPCRs performed on one sample.

Core samples	*nirS*				*nirK*				*nosZ*			
	Core-A		Core-B		Core-A		Core-B		Core-A		Core-B	
	Copies/g	SD [a]	Copies/g	SD	Copies/g	SD	Copies/g	SD	Copies/g	SD	Copies/g	SD
2005.10cm	1.9E+06	1.6E+05	8.5E+05	9.2E+04	3.3E+05	9.5E+04	5.6E+04	2.1E+04	3.6E+05	5.6E+04	2.1E+06	7.9E+04
2005.15cm	3.1E+06	6.9E+05	3.9E+05	3.1E+04	6.0E+05	1.3E+05	6.2E+04	1.4E+04	2.0E+05	1.0E+05	6.5E+05	5.5E+04
2005.30cm	3.0E+06	2.4E+05	1.3E+05	2.1E+04	7.3E+05	6.0E+04	1.3E+04	9.8E+02	8.9E+05	1.0E+05	1.1E+05	2.4E+03
2005.35cm	2.7E+05	6.5E+04	1.6E+04	5.1E+02	2.1E+04	3.7E+03	2.5E+03	1.7E+03	6.7E+04	8.1E+03	1.5E+04	3.3E+03
2005.50cm	5.7E+02	1.6E+02	1.1E+05	2.4E+03	1.1E+03	1.5E+02	1.3E+04	7.2E+02	9.1E+02	6.9E+02	1.2E+05	2.1E+04
2005.55cm	8.2E+02	6.1E+02	1.8E+02	1.1E+02	8.1E+02	1.0E+03	8.3E+02	5.2E+02	1.2E+03	9.8E+01	2.2E+02	1.0E+02
2005.60cm	0	0	1.5E+02	2.8E+00	0	0	5.4E+03	4.3E+03	0	0	1.1E+03	8.1E+02
2005.65cm	0	0	3.8E+02	4.3E+02	0	0	1.4E+03	1.4E+03	3.8E+03	1.1E+03	6.8E+02	1.5E+02
2005.70cm	3.8E+03	1.1E+03	2.5E+02	2.8E+02	1.1E+03	5.1E+01	5.2E+02	4.6E+01	2.0E+03	5.5E+02	1.1E+03	1.4E+03
2010.5cm	3.1E+05	5.0E+04	1.7E+04	2.4E+03	2.8E+04	3.5E+02	3.4E+04	8.3E+02	8.6E+04	1.4E+04	3.1E+04	5.4E+03
2010.10cm	1.3E+05	6.7E+03	1.1E+04	3.7E+02	8.3E+03	1.3E+03	4.1E+03	9.1E+02	3.7E+04	8.4E+03	2.5E+04	6.4E+03
2010.15cm	1.9E+05	9.1E+03	4.4E+04	4.9E+02	2.2E+04	2.1E+03	4.5E+03	3.2E+01	2.4E+05	7.0E+04	6.2E+03	4.3E+01
2010.30cm	6.0E+05	2.9E+04	4.9E+04	1.0E+04	6.2E+04	3.6E+02	1.6E+04	1.4E+04	2.9E+05	9.7E+04	8.5E+04	5.5E+04
2010.40cm	4.0E+05	5.0E+05	3.4E+04	9.1E+02	1.1E+05	1.1E+05	4.3E+03	8.7E+02	1.4E+05	1.1E+05	4.6E+04	1.5E+04
2010.60cm	5.4E+05	4.9E+04	2.3E+04	5.6E+03	4.0E+04	3.3E+03	2.9E+03	2.1E+03	2.4E+05	1.5E+03	1.6E+04	2.7E+03
2011.10cm	4.2E+04	2.4E+03	1.2E+04	1.4E+03	1.9E+03	8.5E+02	1.4E+03	1.4E+03	1.5E+04	3.4E+03	5.0E+03	1.3E+03
2011.15cm	2.5E+02	5.8E+01	4.7E+03	2.1E+03	1.5E+03	6.0E+02	1.2E+03	6.5E+02	1.1E+03	5.9E+02	1.3E+03	1.0E+03
2011.30cm	8.5E+02	4.4E+02	2.7E+03	9.3E+02	1.0E+03	1.7E+02	5.3E+02	4.5E+02	7.8E+02	7.2E+02	8.9E+02	2.2E+02
2011.35cm	3.1E+02	3.3E+01	2.0E+03	4.2E+02	2.4E+03	1.3E+03	7.2E+02	2.4E+02	1.4E+02	6.8E+01	2.2E+02	8.2E+01
2011.60cm	4.4E+02	7.2E+01	4.3E+02	5.9E+01	7.5E+02	7.2E+02	1.1E+03	6.1E+00	1.1E+02	1.4E+01	0	0

[a] SD: Standard deviation

Table S9. Number of reads per sample.

Core samples	Reads	Core samples	Reads
2005.10cm.a	4337	2005.10cm.b	5407
2005.15cm.a	4677	2005.15cm.b	6083
2005.30cm.a	4162	2005.30cm.b	6053
2005.35cm.a	11510	2005.35cm.b	5419
2005.50cm.a	5430	2005.50cm.b	4037
2005.55cm.a	3570	2005.55cm.b	967
2005.60cm.a	4536	2005.60cm.b	4152
2005.65cm.a	11820	2005.65cm.b	5611
2005.70cm.a	4488	2005.70cm.b	6890
2010.10cm.a	6640	2010.10cm.b	5014
2010.15cm.a	7824	2010.15cm.b	4068
2010.30cm.a	8269	2010.30cm.b	1854
2010.40cm.a	7688	2010.40cm.b	4152
2010.5cm.a	6714	2010.5cm.b	4489
2010.60cm.a	7138	2010.60cm.b	3537
2011.10cm.a	20335	2011.10cm.b	6700
2011.15cm.a	16547	2011.15cm.b	8126
2011.30cm.a	8325	2011.30cm.b	8222
2011.35cm.a	8989	2011.35cm.b	9086
2011.60cm.a	8682	2011.60cm.b	7862

Table S10. Taxa that significantly changed over time between pre- and post-WWTP sediment core samples. The taxa are ordered by the effect size, according to a linear regression model where logarithmic relative abundance is predicted based on the core type, year (2005, 2010, and 2011), and depth (10, 15, 30, and 60 cm depths where complete data was available) as fixed effects. The effect size indicates how much the sampling year affects the log abundance according to the model. A small constant (the minimum of the non-zero values) was added to the relative abundances to avoid singularities on zero abundances in taking the logarithm. The analysis was performed with R [13].

Level	Taxon	q-value	Effect size
Phylum	*Chloroflexi*	0.00061	-0.24705
Phylum	*Nitrospirae*	0.22936	0.11436
Phylum	*Firmicutes*	0.04272	-0.11413
Phylum	*Proteobacteria*	0.00001	0.05176
Order	*Anaerolineales*	0.00002	-0.30582
Order	*Lactobacillales*	0.00594	-0.19897
Order	*Clostridiales*	0.02586	-0.17292
Order	*Burkholderiales*	0.00000	0.15111
Order	*Rhodocyclales*	0.17111	-0.14196
Order	*Desulfobacterales*	0.08725	-0.13968
Order	*Xanthomonadales*	0.19946	0.10104

Table S11. Diversity comparisons between different cores based on t-test as performed in R [13]. Significant difference at a q-value < 0.25 is marked as TRUE/FALSE.

Group-1	Group-2	Shannon		Chao1		OTU count	
		q-value	significant	q-value	significant	q-value	significant
2005-A	2005-B	1.000	FALSE	1.0000	FALSE	1.000	FALSE
2005-A	2011-A	0.001	TRUE	0.001	TRUE	0.001	TRUE
2005-A	2011-B	0.254	FALSE	0.805	FALSE	0.678	FALSE
2005-A	2010-A	0.000	TRUE	0.000	TRUE	0.000	TRUE
2005-A	2010-B	0.001	TRUE	0.000	TRUE	0.000	TRUE
2005-B	2011-A	0.000	TRUE	0.000	TRUE	0.000	TRUE
2005-B	2011-B	0.536	FALSE	0.805	FALSE	0.681	FALSE
2005-B	2010-A	0.000	TRUE	0.000	TRUE	0.000	TRUE
2005-B	2010-B	0.001	TRUE	0.000	TRUE	0.000	TRUE
2011-A	2011-B	0.001	TRUE	0.048	TRUE	0.042	TRUE
2011-A	2010-A	0.000	TRUE	0.000	TRUE	0.000	TRUE
2011-A	2010-B	0.000	TRUE	0.000	TRUE	0.000	TRUE
2011-B	2010-A	0.024	TRUE	0.004	TRUE	0.004	TRUE
2011-B	2010-B	0.536	FALSE	0.457	FALSE	0.769	FALSE
2010-A	2010-B	0.001	TRUE	0.000	TRUE	0.000	TRUE

References

1. Muyzer G, De Waal EC, Uitterlinden AG. Profiling of complex microbial populations by denaturing gradient gel electrophoresis analysis of polymerase chain reaction-amplified genes coding for 16S rRNA. *Appl Environ Microbiol* **59**, 695-700 (1993).

2. Smits THM, Devenoges C, Szynalski K, Maillard J, Holliger C. Development of a real-time PCR method for quantification of the three genera *Dehalobacter*, *Dehalococcoides*, and *Desulfitobacterium* in microbial communities. *J Microbiol Methods* **57**, 369-378 (2004).

3. Dar SA, Yao L, Van Dongen U, Kuenen JG, Muyzer G. Analysis of diversity and activity of sulfate-reducing bacterial communities in sulfidogenic bioreactors using 16S rRNA and dsrB genes as molecular markers. *Appl Environ Microbiol* **73**, 594-604 (2007).

4. Wilms R, Sass H, Köpke B, Cypionka H, Engelen B. Methane and sulfate profiles within the subsurface of a tidal flat are reflected by the distribution of sulfate-reducing bacteria and methanogenic archaea. *FEMS Microbiol Ecol* **59**, 611-621 (2007).

5. Zheng Y*, et al.* Diversity, abundance, and activity of ammonia-oxidizing bacteria and archaea in Chongming eastern intertidal sediments. *Appl Microbiol Biotechnol*, 1-13 (2012).

6. Michotey V, Méjean V, Bonin P. Comparison of methods for quantification of cytochrome cd1-denitrifying bacteria in environmental marine samples. *Appl Environ Microbiol* **66**, 1564-1571 (2000).

7. Throbäck IN, Enwall K, Jarvis A, Hallin S. Reassessing PCR primers targeting *nirS*, *nirK* and *nosZ* genes for community surveys of denitrifying bacteria with DGGE. *FEMS Microbiol Ecol* **49**, 401-417 (2004).

8. Henry S, Baudoin E, López-Gutiérrez JC, Martin-Laurent F, Brauman A, Philippot L. Quantification of denitrifying bacteria in soils by nirK gene targeted real-time PCR. *J Microbiol Methods* **59**, 327-335 (2004).

9. Henry S, Bru D, Stres B, Hallet S, Philippot L. Quantitative detection of the nosZ gene, encoding nitrous oxide reductase, and comparison of the abundances of 16S rRNA, narG, nirK, and nosZ genes in soils. *Appl Environ Microbiol* **72**, 5181-5189 (2006).

10. van den Bogert B, de Vos WM, Zoetendal EG, Kleerebezem M. Microarray analysis and barcoded pyrosequencing provide consistent microbial profiles depending on the source of human intestinal samples. *Appl Environ Microbiol* **77**, 2071-2080 (2011).

11. Daims H, Brühl A, Amann R, Schleifer KH, Wagner M. The domain-specific probe EUB338 is insufficient for the detection of all bacteria: Development and evaluation of a more comprehensive probe set. *Systematic and Applied Microbiology* **22**, 434-444 (1999).

12. Lane D. 16S/23S rRNA sequencing. In Nucleic Acid Techniques in Bacterial Systematics. Stackebrandt, E., and Goodfellow, M. (eds). New York, USA: Wiley, pp. 115–148., (1991).

13. R Core Team. R: A language and environment for statistical computing. (ed^(eds). R Foundation for Statistical Computing (2013).
